# Pyrrothiogatain acts as an inhibitor of GATA family proteins and inhibits Th2 cell differentiation *in vitro*

**DOI:** 10.1038/s41598-019-53856-1

**Published:** 2019-11-22

**Authors:** Shunsuke Nomura, Hirotaka Takahashi, Junpei Suzuki, Makoto Kuwahara, Masakatsu Yamashita, Tatsuya Sawasaki

**Affiliations:** 1 Proteo-Science Center (PROS), Ehime University, 3 Bunkyo-cho, Matsuyama, Ehime 790-8577 Japan; 2 Department of Immunology, Graduate School of Medicine, Ehime University, Shitsukawa, Toon, 791-0295 Ehime Japan

**Keywords:** Transcription factors, Screening, High-throughput screening, T-helper 2 cells

## Abstract

The transcription factor GATA3 is a master regulator that modulates T helper 2 (Th2) cell differentiation and induces expression of Th2 cytokines, such as IL-4, IL-5, and IL-13. Th2 cytokines are involved in the protective immune response against foreign pathogens, such as parasites. However, excessive production of Th2 cytokines results in type-2 allergic inflammation. Therefore, the application of a GATA3 inhibitor provides a new therapeutic strategy to regulate Th2 cytokine production. Here, we established a novel high-throughput screening system for an inhibitor of a DNA-binding protein, such as a transcription factor, and identified pyrrothiogatain as a novel inhibitor of GATA3 DNA-binding activity. Pyrrothiogatain inhibited the DNA-binding activity of GATA3 and other members of the GATA family. Pyrrothiogatain also inhibited the interaction between GATA3 and SOX4, suggesting that it interacts with the DNA-binding region of GATA3. Furthermore, pyrrothiogatain significantly suppressed Th2 cell differentiation, without impairing Th1 cell differentiation, and inhibited the expression and production of Th2 cytokines. Our results suggest that pyrrothiogatain regulates the differentiation and function of Th2 cells via inhibition of GATA3 DNA binding activity, which demonstrates the efficiency of our drug screening system for the development of novel small compounds that inhibit the DNA-binding activity of transcription factors.

## Introduction

Transcription factors are key molecules that regulate gene expression and cell fate in response to cell signalling stimuli from extracellular environments^1,2^. As a final step of multiple signal transductions, transcription factors regulate gene expression via nuclear translocation, dimerization, and DNA binding on the target regulatory regions in the genome. In the case of a pharmacological target of transcription factors, the direct inhibition of these events facilitates drug development^3^. The main biochemical properties of transcription factors are DNA binding and protein-protein interaction. However, it is difficult to establish a drug screening system to generate inhibitors against transcription factors because the recombinant expression and purification of bioactive transcription factors is challenging because, without their partner proteins or target DNA, they generally have an unstable structure^3,4^. In addition, transcription factors, except for the nuclear receptor family, lack a deep hydrophobic pocket that allows access to a small molecular compound which modulates its biological function^4^. Therefore, it remains a challenge to develop an inhibitor against transcription factors, which have been regarded as an efficient target for therapy.

After recognizing foreign antigens, naive CD4^+^ T cells differentiate into several effector helper T cell subsets including Th1, Th2, Th17, and iTreg, which play key roles in adaptive immune responses; but they have the potential to drive immune-mediated diseases^5,6^. The pathology of allergic asthma, which is a Th2 cytokine-mediated disorder, is characterized by eosinophilia, goblet cell hyperplasia, and airway smooth muscle contraction^7^. These pathological events are due to the excessive differentiation and activation of Th2 cells^8^, which are a major source of Th2 cytokines, such as IL-4, IL-5, and IL-13^9^. Therefore, a strategy that modulates the differentiation and function of Th2 cells will lead to a novel therapeutic strategy for allergic disorders.

The GATA (GATA-binding protein) family consists of six members from GATA1 to GATA6. They contain a transactivation domain in the N-terminal region and a zinc-finger DNA-binding domain in the C-terminal region, and bind to the specific DNA sequence (A/T)-GATA-(A/G). GATA3 has been identified as a master regulator that controls Th2 cell differentiation and the production of Th2 cytokines by binding to a broad range of Th2 cytokine gene locus^10,11^, whereas GATA3 suppresses Th1 cell differentiation by blocking transcription of Th1-specific genes^12–16^. Among the Th2 cytokine genes, IL-5 and IL-13 are directly regulated by the binding of GATA3 with the corresponding promoter region^5,17^. Importantly, the upregulation of GATA3 has been reported in allergic inflammatory cells in allergy patients^18,19^ and is considered an attractive therapeutic target for ameliorating allergic inflammation^8^.

In this study, we established a high-throughput inhibitor screening system for transcription factors using GATA3 as a model. The inhibitor screening system specifically identified factors inhibiting the DNA-binding activity of transcription factors and facilitated the development of a novel GATA family inhibitor, pyrrothiogatain. Our results showed pyrrothiogatain inhibited the DNA-binding activity of GATA3. Furthermore, pyrrothiogatain suppressed *in vitro* Th2 cell differentiation and the secretion of Th2 cytokines without impairing Th1 cell differentiation.

## Results

### Establishment of a high-throughput assay to detect a DNA–protein interaction

**Figure 1:**
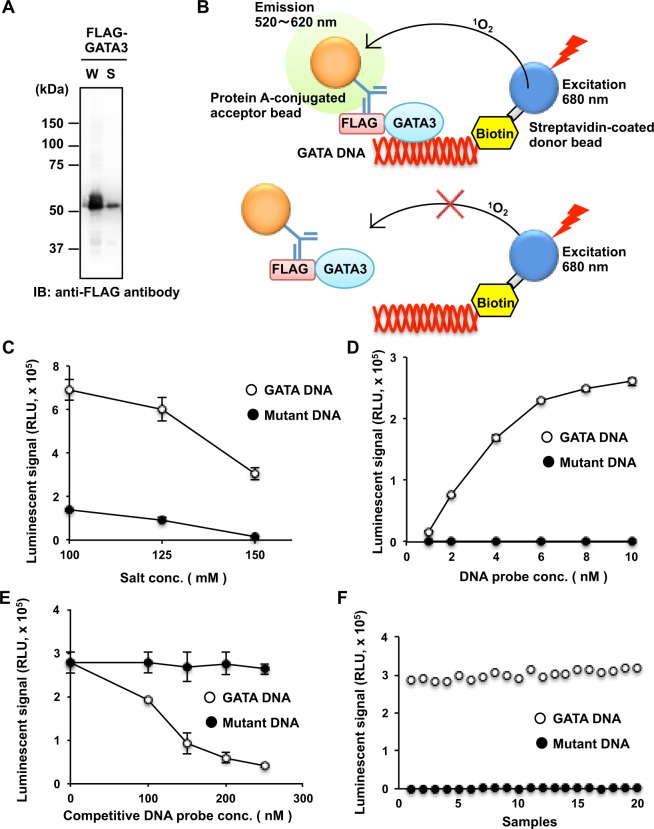
Establishment of the high-throughput assay system to directly detect a DNA–protein interaction. (**A**) Immunoblot analysis of FLAG-tagged recombinant GATA3 (FLAG-GATA3) synthesized by the wheat cell-free system. The whole translational mixture (W) and the supernatant (S), obtained after centrifugation, were analysed using anti-FLAG M2 antibody. (**B**) A schematic diagram of the high-throughput biochemical DNA-binding assay system to detect the direct binding between GATA3 and its target DNA. When FLAG-tagged GATA3 binds the DNA labelled with biotin at the 5 prime-terminal, AlphaScreen beads generate luminescent signal. (**C**) The binding assay of GATA3 with its consensus DNA-binding motif. The binding assay between the crude translation mixture of FLAG-tagged GATA3 (1 µL) and biotinylated DNA (10 nM) was performed in the presence of various concentrations of NaCl (100 to 150 mM). An oligonucleotide with a mutated GATA-binding site was used as control for this assay. (**D**) The binding assay as described in (**C**) was performed in the presence of indicated concentrations of biotinylated DNA. A reaction mixture containing FLAG-tagged GATA3 and 150 mM NaCl prepared under the same conditions as those in (**C**) was mixed with 1 to 10 nM biotinylated DNA. (**E**) Competition assay with non-labelled GATA consensus DNA. The binding assay with the same conditions as those in (**D**) was mixed with biotinylated DNA (4 nM) and non-labelled GATA consensus DNA (0 to 250 nM) as a control. (**F**) Validation of the quality of the binding assay using AlphaScreen. The ‘Z’ factor was calculated from the binding reaction of GATA3 with the GATA consensus DNA (positive control, n = 20) or its GATA-binding site mutant (negative control, n = 20). In (**C–E**), all data are expressed as individual points of three independent experiments with error bars indicating standard deviation.

Previously, we developed a drug screening system to generate an inhibitor against a protein-protein interaction based on a wheat cell-free system and AlphaScreen technology^20,21^, which is a high-throughput luminescence-based binding assay. We identified an NF-κB inhibitor (DANFIN)^20^ and two agonists for abscisic acid receptor (JFA1 and JFA2)^21^. In this study, we attempted to construct a drug screening system for the development of inhibitors against a DNA-protein interaction using the cell-free based system. As a model, we selected the GATA3 transcription factor because GATA3 is known as the master regulator for Th2 cell differentiation and production of Th2 cytokines^10,11^ and GATA3-binding to its DNA sequence has already been reported^11^. To determine the functions of the GATA3 protein, we synthesized the recombinant full-length GATA3 protein with an N-terminal FLAG tag using the wheat cell-free system. The levels of GATA3 in the whole translational mixture (W) and the supernatant (S), obtained after centrifugation of the former, were determined by immunoblot analysis (Fig. 1A), indicating that the recombinant full-length GATA3 was synthesized as a soluble form.

We used AlphaScreen technology^20,21^ to detect the direct binding between GATA3 and its target DNA (TGATAA) labelled with biotin (Fig. 1B). We performed the binding assay using recombinant FLAG-tagged full-length GATA3 and the biotinylated GATA-consensus DNA (GATA DNA) or the negative control DNA mutated in the GATA-binding motif (mutant DNA) under various NaCl concentrations (100 to 150 mM). A high luminescence signal was obtained by the binding between GATA3 and the target DNA (Fig. 1C). A considerable luminescence signal due to non-specific binding of GATA3 with the negative control DNA was also observed in the presence of 100 mM and 125 mM NaCl, whereas the luminescence signal of the negative control was disrupted in the presence of 150 mM NaCl. In addition, the binding assay was performed under various concentrations of GATA DNA, for which the luminescence signal intensified in a dose-dependent manner (Fig. 1D). In contrast, the negative control DNA produced a luminescence signal (<1500) of very low intensity comparable to the background intensity level. These results indicated that this binding system could detect the interaction between specific DNA and the GATA3 protein.

To further validate whether the binding assay could detect the inhibition of the interaction between biotinylated DNA and the GATA3 protein, a competition assay was performed using supplementary non-labelled DNA with the same sequence. As a result, a dose-dependent decrease in the intensity of the luminescence signal was observed by supplementation with non-labelled GATA consensus DNA (Fig. 1E). In contrast, the DNA binding activity of GATA3 was not influenced by mutant DNA supplementation. These results suggested that this assay could detect the inhibition of the GATA3–DNA interaction.

Next, we investigated whether the assay was suitable for high-throughput screening by 20-independent positive reactions and negative reactions, and a Z’ factor, known as indicator of the accuracy and quality of high-throughput screening, that was calculated for the validation. The Z’ factor was found to be 0.88 (Fig. 1F), indicating that this cell-free assay for the protein-DNA interaction could be used for high-throughput screening.

### Identification of a GATA3 inhibitor using the wheat cell-free drug screening system

**Figure 2:**
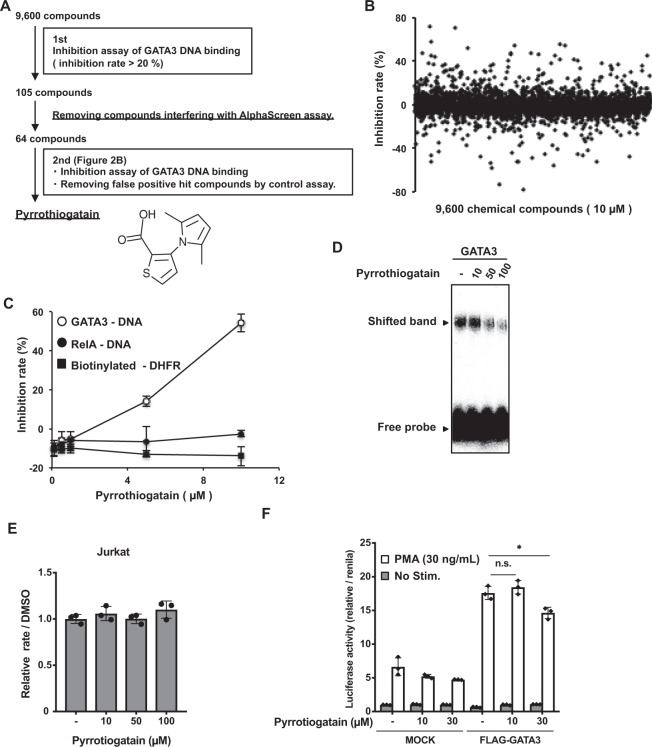
Identification of a GATA3 inhibitor using the wheat cell-free drug screening system. (**A**) A flow chart of the high-throughput screening to identify the 3-(2,5-dimethyl-1H-pyrrol-1-yl)thiophene-2-carboxylic acid that inhibits the DNA-binding activity of GATA3. The 3-(2,5-dimethyl-1H-pyrrol-1-yl)thiophene-2-carboxylic acid was named pyrrothiogatain (pyrrole and thiofuran containing GATA3 inhibitor). (**B**) The results of the first screen using 9,600 compounds. In the assay, each chemical compound was used at a final concentration of 10 µM. (**C**) The results of the second screening that show the inhibition rate of pyrrothiogatain. The inhibition assay of GATA3 DNA-binding activity was performed in the presence of various concentrations of pyrrothiogatain (0 to 10 µM). Biotinylated FLAG-peptide was used as a control to measure the interference of compounds in the AlphaScreen assay. In addition, a binding assay of RelA and its target DNA was used as control for GATA3. (**D**) Electrophoretic mobility shift assay (EMSA) of GATA3 with its consensus motif containing oligonucleotide in the presence of various concentrations of pyrrothiogatain (0 to 100 µM). GATA3 consensus oligonucleotide labelled with ^32^P was detected by autoradiography. (**E**) MTS assay to confirm the cytotoxicity of pyrrothiogatain. Jurkat cells were cultured with various concentrations of pyrrothiogatain (0 to 100 µM) for 3 days and subjected to MTS assay. (**F**) HEK293T cells were transfected with firefly luciferase reporter for the *Il-5* promoter plus renilla luciferase reporter in the presence (+) or absence (−) of GATA3-expressing plasmid. Then, the cells were left unstimulated (Med.) or stimulated (Stim.) with the phorbol ester PMA (30 ng/mL) in the presence of pyrrothiogatain (0 to 30 µM). The luciferase activity is presented relative to renilla luciferase activity. In (**B,D,E**), all data are expressed as individual points of three independent experiments with error bars indicating standard deviation.

We performed high-throughput screening using the assay shown in Fig. 1 to identify inhibitors of GATA3 DNA binding activity. The results of drug screening are summarized as a flow chart in Fig. 2A. We identified 105 candidate compounds that showed an inhibition rate >20% at a compound concentration of 10 µM in the first screening of 9,600 compounds (Fig. 2B). We performed a second screen to exclude the false-positive compounds. As shown in Fig. 2C, one compound was identified that specifically inhibited GATA3–DNA binding without interfering with both the AlphaScreen assay and the RelA-DNA interaction. We identified this compound (3-(2,5-dimethyl-1H-pyrrol-1-yl)thiophene-2-carboxylic acid) as a pyrrothiogatain (pyrrole and thiofuran containing GATA3 inhibitor) (Fig. 2A). The inhibitory activity of pyrrothiogatain for the GATA3-DNA interaction was validated by electrophoretic mobility shift assay (EMSA). The intensity of the shifted bands generated by GATA3-DNA binding was decreased in a pyrrothiogatain dose-dependent manner (50 to 100 µM) (Fig. 2D), indicating that pyrrothiogatain inhibited the GATA3–DNA interaction.

Next, cytotoxicity was performed to assess whether pyrrothiogatain could be used in a cell-based assay. Jurkat cells were cultured in the presence of various concentrations of pyrrothiogatain (0 to 100 µM) for 3 days and cell viability was determined by MTS assay. As shown in Fig. 2E, pyrrothiogatain did not influence cell viability. GATA3 induces the transcription of the *IL-5* gene through the direct binding of the GATA consensus motif on the IL-5 promoter^5^. We therefore investigated whether pyrrothiogatain inhibited GATA3-dependent IL-5 promoter activation using a luciferase reporter assay with the IL-5 promoter^22,23^. As shown in Fig. 2F, pyrrothiogatain significantly suppressed GATA3-dependent transcriptional activation of the IL-5 promoter, suggesting that pyrrothiogatain inhibited DNA binding activity of GATA3 in the cells. Taken together, these results indicated that pyrrothiogatain, obtained from the wheat cell-free based screening, inhibited the function of GATA3.

### Effect of pyrrothiogatain on the DNA-binding activity of other GATA family proteins

**Figure 3:**
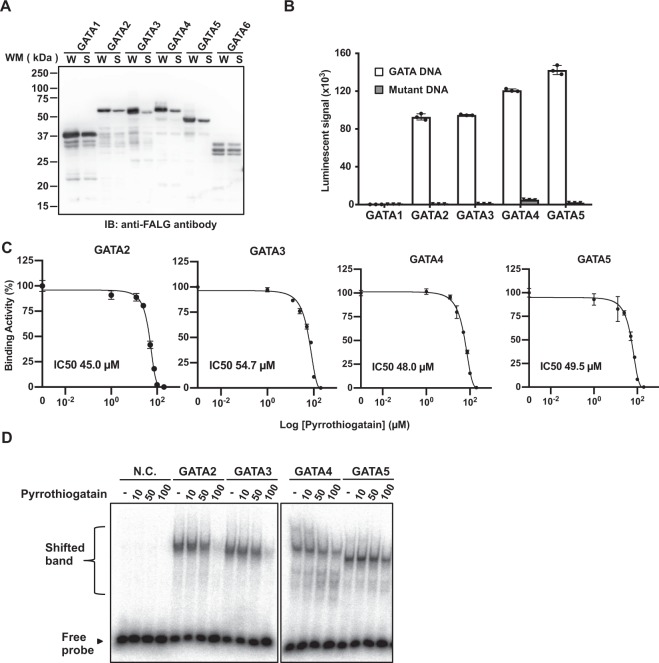
Effect of pyrrothiogatain on the DNA-binding activity of GATA family proteins. (**A**) Immunoblot analysis of FLAG-tagged recombinant GATA family proteins (GATA1-GATA6) synthesized by the wheat cell-free system. The whole translational mixture (W) and the supernatant (S) were analysed using an anti-FLAG M2 antibody. (**B**) The AlphaScreen assay to detect the binding between the GATA family and DNA containing the GATA consensus binding sequence using the same protocol as that in Fig. 1F. (**C**) The inhibition assays for the DNA-binding activity of GATA family proteins (GATA2-GATA5) were performed in the presence of various concentrations of pyrrothiogatain (0 to 200 µM). The binding activity is represented by the relative AlphaScreen signal. (**D**) The results of EMSA of the binding GATA family proteins (GATA2-GATA5) in the presence of pyrrothiogatain (0 to 100 µM). GATA binding to the DNA labelled with ^32^P was detected by autoradiography. In (**B,C**), all data are expressed as individual points of three independent experiments with error bars indicating standard deviation.

We next assessed the inhibitory effect of pyrrothiogatain on the DNA-binding activities of other GATA family proteins using the cell-free system and AlphaScreen technology. Six GATA proteins from GATA1 to GATA6 have been reported^11^, and we tried to synthesize their full-length proteins using the cell-free system. GATA proteins, except for GATA6, were successfully synthesized as a soluble form (Fig. 3A), and four GATA proteins, GATA2 to GATA5, exhibited comparable DNA-binding activity (Fig. 3B). Since the GATA1 protein did not bind to the same DNA probe, it was excluded from further analysis. Pyrrothiogatain inhibited the DNA-binding activity of GATA2 to GATA5 (Fig. 3C), with an approximate IC50 value of 50 µM. Similar inhibitory results were obtained by EMSA (Fig. 3D). These data suggest that pyrrothiogatain acts as a pan-inhibitor of GATA family proteins.

### Pyrrothiogatain inhibited the GATA3-SOX4 interaction

**Figure 4:**
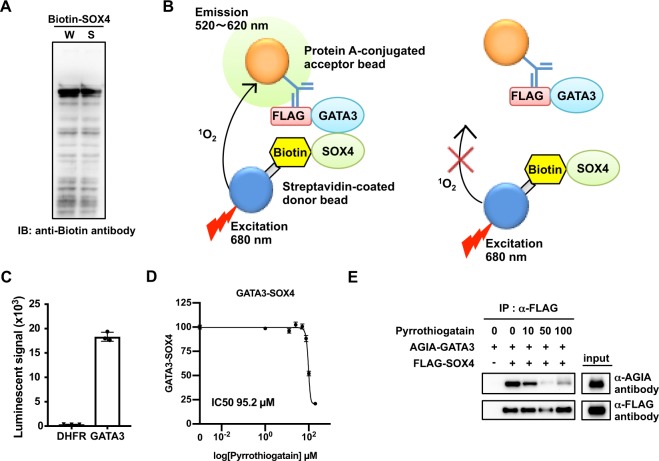
Pyrrothiogatain inhibits the GATA3–SOX4 interaction. (**A**) Immunoblot analysis of biotin fused recombinant SOX4 (Biotin-SOX4) synthesized by the wheat cell-free system. The whole translational mixture (W) and the supernatant (S) were analysed using an anti-biotin antibody. (**B**) A schematic diagram of the binding assay to detect the direct binding between GATA3 and SOX4. When FLAG-tagged GATA3 binds with biotin fused SOX4, AlphaScreen beads generate a luminescent signal. (**C**) The binding assay of GATA3 and SOX4. The luminescence signals were detected by AlphaScreen technology. FLAG-DHFR was used as a control. (**D**) The inhibition assay of the binding of GATA3 and SOX4 in the presence of various concentrations of pyrrothiogatain (0 to 100 µM). The binding activity was calculated from the AlphaScreen signal. (**E**) The binding of recombinant GATA3 with SOX4 in the presence of pyrrothiogatain (0 to 100 µM) was assessed by immunoprecipitation assay. AGIA-tagged GATA3 and FLAG-tagged SOX4 were synthesized by the wheat cell system and subjected to an immunoprecipitation assay using an anti-FLAG-affinity agarose gel. In (**C,D**), all data are expressed as individual points of three independent experiments with error bars indicating standard deviation.

The GATA3 protein has been reported to directly interact with SOX4^22^. We next investigated whether pyrrothiogatain interrupted the interaction between GATA3 and SOX4. As shown in Fig. 4A, an immunoblotting analysis revealed that an N-terminal biotinylated full-length SOX4 protein was synthesized by the wheat cell-free system. Interaction between biotin-labelled SOX4 and FLAG-GATA3 was analysed by the AlphaScreen system (Fig. 4B). The interaction indicated that biotinylated SOX4 directly interacted with FLAG-GATA3 (Fig. 4C), but not with FLAG-DHFR, a negative control. A pyrrothiogatain dose-dependent decrease in GATA3-SOX4 interaction was detected using AlphaScreen (Fig. 4D) and immunoprecipitation (Fig. 4E). These results suggested that pyrrothiogatain also inhibited the protein-protein interaction of GATA3.

### Pyrrothiogatain inhibited Th2 cell differentiation and the production of Th2 cytokines

It is well established that GATA3 induces Th2 cell differentiation^11^. Since pyrrothiogatain exhibited no effect on the viability of Jurkat T cells (Fig. 2E), we investigated whether pyrrothiogatain inhibited Th2 cell differentiation. Total CD4^+^ T cells were isolated and cultured under IL-2 conditions in the presence or absence of pyrrothiogatain for five days, and then the Th2 cell differentiation status was determined using intracellular staining. The generation of IL-4-, IL-5-, and IL-13-producing cells was repressed by the treatment of pyrrothiogatain (Supplementary Fig. 1A), whereas the number of Th1 cells producing IFN-γ was increased. In addition, decreased production and mRNA expression of IL-4, IL-5, IL-10, and IL-13 were confirmed using ELISA (Supplementary Fig. 1B) and RT-qPCR (Supplementary Fig. 1C). Increased production and mRNA expression of IFN-γ were also detected. The expression of GATA3 was not influenced at either the mRNA or protein level (Supplementary Fig. 1C,D). A previous report indicated that GATA3 may silence the expression of *Tbx21*, a master regulator of Th1 cells, by directly binding the *Tbx21* locus^16^. As expected, the expression of *Tbx21* was increased after treatment with pyrrothiogatain (Supplementary Fig. 1C).

**Figure 5:**
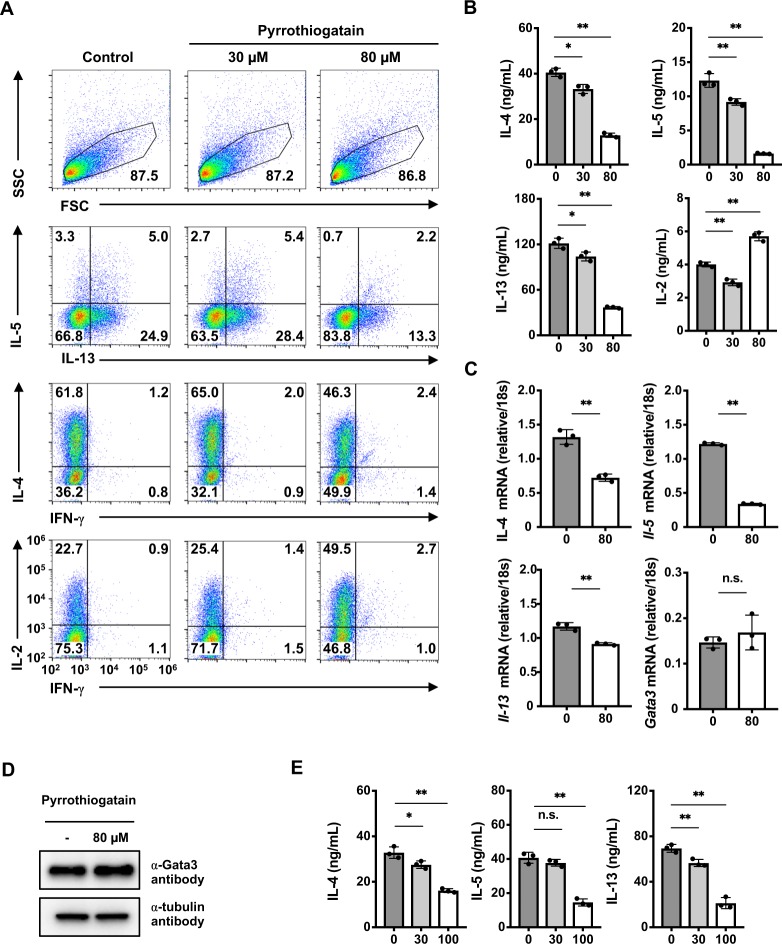
Pyrrothiogatain inhibits Th2 cell differentiation and production of Th2 cytokines. (**A**) Intracellular staining of IL-5/IL-13, IL-4/IFN-γ, and IL-2/IFN-γ in naive CD4^+^ T cells cultured under Th2 conditions in the presence or absence of pyrrothiogatain (30 and 80 µM) for five days. (**B**) Cytokine production induced in the pyrrothiogatain treated Th2 cells shown in panel (A) was determined by ELISA. (**C**) Quantitative RT-PCR analysis of the pyrrothiogatain-treated Th2 cells shown in panel (A). (**D**) Immunoblot analysis of GATA3 in naive CD4^+^ T cells cultured under Th2 conditions in the presence or absence of pyrrothiogatain (80 µM) for 5 days. (**E**) Cytokine production from Th2 cells stimulated with immobilized anti-TCRβ mAb for 16 h in the presence or absence of pyrrothiogatain (0 to 100 µM). The amounts of IL-4, IL-5 and IL-13 in the culture supernatants were determined by ELSA. In (**B**), (**C**) and (**E**), all data are expressed as individual points of three independent experiments with error bars indicating standard deviation.

To clarify the effects of pyrrothiogatain on Th2 cell differentiation, naive CD4 T cells were purified and cultured under Th2 conditions in the presence or absence of pyrrothiogatain for 5 days, and then the Th2 cell differentiation status was determined by intracellular staining. The generation of IL-4-, IL-5-, and IL-13-producing Th2 cells was repressed by treatment with pyrrothiogatain (Fig. 5A). Decreased production and mRNA expression of IL-4, IL-5 and IL-13 were confirmed using ELISA and RT-qPCR, respectively (Fig. 5B,C). However, the expression of GATA3 was not influenced at either the mRNA or protein level (Fig. 5C,D). The generation of IFN-γ and IL-2 producing Th1 cells under Th1 conditions was modestly increased by treatment with pyrrothiogatain (supplementary Fig. 2A,B). These results indicated that pyrrothiogatain selectively inhibited Th2 cell differentiation. Next, we treated *in vitro* differentiated Th2 cells with pyrrothiogatain to test the effect of pyrrothiogatain on Th2 cytokine production. Total CD4^+^ cells were cultured under Th2 conditions for 5 days, and then the Th2 cells were restimulated in the presence or absence of pyrrothiogatain for 16 h. As shown in Fig. 5E, IL-4, IL-5 and IL-13 production was decreased by pyrrothiogatain treatment. Finally, we investigated the effects of the administration of pyrrothiogatain in a mouse model of OVA-induced type 2 airway inflammation. We expected that pyrrothiogatain might exhibit a positive therapeutic effect against Th2-dependent allergic inflammation. However, the administration of pyrrothiogatain did not significantly decrease the number of inflammatory cells in the bronchoalveolar lavage (BAL) fluid of OVA-induced allergic mice (Supplementary Fig. 3A,B).

Taken together, these results indicated that pyrrothiogatain inhibited Th2 cell differentiation and the production of Th2 cytokines *in vitro*, without influencing the expression of GATA3.

### Pyrrothiogatain inhibited GATA3 binding to Th2 cytokine gene locus

**Figure 6:**
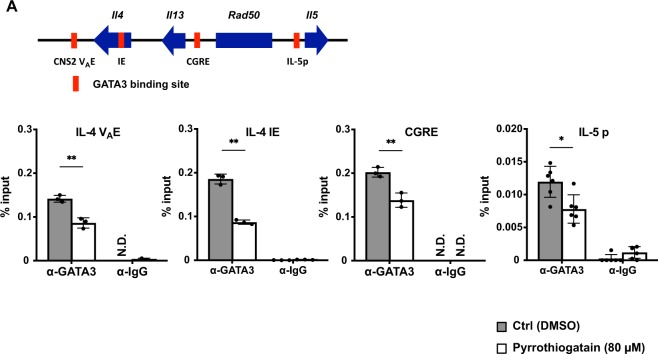
Pyrrothiogatain inhibits GATA3 DNA-binding to Th2 cytokine gene locus. (**A**) The GATA3 binding sites in Th2 cells are shown as a schematic diagram. Chromatin immunoprecipitation analysis of GATA3 in naive CD4^+^ T cells cultured under Th2 conditions in the presence or absence of pyrrothiogatain (80 µM) for 2 days. All data are expressed as mean values of three independent experiments with error bars indicating standard deviation.

GATA3 has been reported to bind to Th2 cytokine gene locus, such as IL-4 V_A_ enhancer^24^, IL-4 intron enhancer^25^, conserved Gata3-response element (CGRE)^26^ and IL-5 promoter^27^ regions in Th2 cells. We investigated whether pyrrothiogatain inhibited GATA3 binding to Th2 cytokine gene locus. Naive CD4^+^ T cells were cultured under Th2 conditions in the presence or absence of 80 µM pyrrothiogatain for 2 days, and then the cells were subjected to chromatin immunoprecipitation with an anti-GATA3 antibody. As shown in Fig. 6A, pyrrothiogatain repressed GATA3 binding to the IL-4 V_A_ enhancer, IL-4 intron enhancer, CGRE and IL-5 promoter regions. These results indicated that pyrrothiogatain inhibited GATA3 DNA-binding to Th2 cytokine gene locus in Th2 cells.

## Discussion

In this study, we established a drug screening system for the development of inhibitors against transcription factors using a cell-free based system. Although several studies have reported the development of various high-throughput inhibitor screens targeting transcription factors^28–30^, our screening system exhibited several advantages. In many cases of pharmacological targeting of transcription factors, methodological problems arise during both the biochemical and cell-based assays. In the case of the biochemical assay, it is difficult to purify transcription factors with DNA-binding activity and to validate that activity with several compounds to determine their biochemical function^3,4^, because many transcription factors harbour unstable structural regions without their partner protein or target DNA^4^. In addition, the GATA3 DNA-binding domain contains a flexible linker with an unstructured region^31^. For cell-based drug screening against transcription factors, a luciferase-reporter assay-based screen has been employed with transcriptional activity as an indicator^30^. However, cell-based assays have a high possibility of developing unexpected compounds, indirectly influencing the function of transcription factors^3^. Our wheat cell-free drug screening system does not demand protein purification and makes it possible to detect the DNA-binding activity of transcription factors directly. Therefore, our screening system is useful for the development of chemical compounds targeting transcription factors.

Using the newly developed screening system, we identified pyrrothiogatain, which inhibited the DNA-binding activity of GATA3, from a chemical library of 9,600 small compounds. Pyrrothiogatain suppressed Th2 cell differentiation and production of Th2 cytokines (Fig. 5A,B) without any influence on GATA3 expression at the mRNA and protein levels (Fig. 5C,D). During Th1 cell differentiation, GATA3 is known to repress IFN-γ production and Th1-specific gene expression^12–16^. Since pyrrothiogatain promoted gene expression and production of IFN-γ (Supplementary Fig. 1), these results also strongly suggested the inhibition of GATA3 function in cells. In addition, genome-wide analysis of GATA3-binding sites indicates that GATA3 may be involved in silencing of *Tbx21* expression by directly binding the *Tbx21* locus^16^. Our results showed that the *Tbx21* expression was induced after treatment with pyrrothiogatain (Supplementary Fig. 1C). Therefore, these results indicated that pyrrothiogatain acts as a regulatory compound against Th-subset differentiation and inhibits Th2 cell differentiation, which simultaneously leads to Th1 cell differentiation.

GATA3 has been reported to bind to Th2 cytokine gene locus, such as the IL-4 V_A_ enhancer^24^, IL-4 intron enhancer^25^ and CGRE^26^. The IL-4 V_A_ enhancer and IL-4 intron enhancer are regulatory regions for IL-4 production as GATA3 binding sites. In addition, the CGRE is important to induce IL-13 production via GATA3 binding. In this study, we confirmed that pyrrothiogatain suppressed GATA3 binding to these Th2 cytokine gene regions. The result indicated the pyrrothiogatain inhibited Th2 cell differentiation via repression of GATA3 binding to Th2 cytokine gene locus in cells.

As a GATA3 inhibitor targeting mRNA, the DNA enzyme (DNAzyme) cleaving GATA3 mRNA, has been developed for therapy of allergic asthma^32,33^. We expected that pyrrothiogatain might exhibit a positive therapeutic effect against Th2-dependent allergic inflammation. However, the administration of pyrrothiogatain did not significantly decrease the number of inflammatory cells in the bronchoalveolar lavage (BAL) fluid of OVA-induced allergic mice (Supplementary Fig. 3A,B). This result indicated that pyrrothiogatain is required to improve the inhibitory activity, affinity to GATA3 and/or bioavailability to ameliorate Th2-dependent allergic inflammation.

We investigated whether pyrrothiogatain exhibited inhibitory effects against the DNA-binding activity of GATA family proteins. AlphaScreen and EMSA analysis showed that pyrrothiogatain suppressed the DNA-binding activity of GATA2-GATA5. These results indicated that pyrrothiogatain influenced the DNA-binding domain of the GATA family, since the DNA-binding domain shows high homology of amino acid sequence among the GATA family. In addition, pyrrothiogatain also inhibited the interaction between GATA3 and SOX4, possibly via interaction with the DNA-binding domain of GATA3^22^. Although the inhibitory effects of pyrrothiogatain could not be evaluated against the DNA-binding activity of GATA1 and GATA6, our experimental results indicated a possibility that pyrrothiogatain inhibited the DNA-binding activity of all GATA family proteins.

The overexpression and dysfunction of transcription factors lead to several human diseases, such as cancer and autoimmune diseases^1^. Therefore, identification of a transcription factor that regulates cell differentiation and fate through regulation of gene expression could be considered as a potential pharmacological target^34^. Instead of controlling transcription factors, regulation of gene expression has also been performed as a therapeutic strategy through the inhibition of upstream signal transduction. However, the pharmacological strategy targeting the signal transducing protein exhibits a lack of specificity and has the potential to generate off-target harmful events^3^. Therefore, development of the drugs directly interacting with transcription factors seems an important issue. In summary, we developed the wheat cell-free drug screening system suitable for development of inhibitors against the DNA-binding activity of transcription factors. Our drug screening system led to the development of a GATA family inhibitor pyrrothiogatain, which regulates Th-subset differentiation, indicating the efficiency of our drug screening system for the development of novel small compounds that inhibit the DNA-binding activity of transcription factors.

## Materials and Methods

### Plasmid construction

The open reading frame of cDNA clones obtained from the Mammalian Gene Collection was subcloned into the pEU-based expression vector for the wheat cell-free protein synthesis system or into pcDNA3.1 for mammalian cell expression.

### Wheat cell-free protein synthesis and immunoblot analysis

*In vitro* transcription and translation were performed by the bilayer method using the WEPRO1240 expression kit (Cell-Free Sciences), according to the manufacturer’s instructions. Biotin labelling was carried out by the method described previously^35^. Specifically, during translation, biotin ligase (BirA), synthesized by a wheat cell-free system, was added to the bottom layer and incubated in the presence of 0.5 μM D-biotin (Nacalai Tesque). The whole lysate and supernatant after its centrifugation were analysed by immunoblotting using anti-FALG-HRP antibody (M2, Cat#A8592, Sigma, 1:4000) and anti-AGIA-HRP^36^.

### DNA-binding assay using AlphaScreen technology

For the protein-DNA binding assay on a 384-well AlphaPlate (Perkin Elmer), 10 μL of bait protein mixture containing 1 µL FLAG-tagged protein in AlphaScreen Buffer (100 mM Tris-HCl (pH 8.0), 150 mM NaCl, 0.1% Tween 20, and 1 mg/mL BSA) and 5 µL biotinylated oligonucleotide (0–10 µM final concentration) in AlphaScreen Buffer were dispended into the AlphaPlate. Then, the plate was incubated at 26 °C for 1 h. After incubation, 10 μL of the detection mixture containing 0.4 µg/mL Anti-DYKDDDDK antibody (1E6, Wako), 0.1 μL streptavidin-conjugated AlphaScreen donor beads, and 0.1 μL protein A-conjugated AlphaScreen acceptor beads in AlphaScreen Buffer was mixed and incubated at 26 °C for 1 h, and then, the AlphaScreen signal was detected using an EnVision device with the AlphaScreen signal detection program (PerkinElmer).

The following oligonucleotides were used:

GATA3 consensus DNA, 5′-biotin-CACTTGATAACAGAAAGTGATAACTCT-3′;

MT, 5′-biotin-CACTTCTTAACAGAAAGTCTTAACTCT-3′.

### Drug screening and validation

For the AlphaScreen-based chemical library screening, we used a diverse set of 9,600 synthesized chemicals established by the Drug Discovery Initiative (The University of Tokyo, Tokyo, Japan). All chemical compounds (10 mM) were dissolved in DMSO, and were pre-dispensed into a 384-well Alphaplate (250 nL/well). Ten microliters of bait protein mixture containing 1 µL of FLAG-tagged GATA3 protein in AlphaScreen Buffer was dispensed into the Alphaplate-384 plate (Cat# 6005350) using a FlexDrop dispenser (PerkinElmer), and 5 µL biotinylated DNA (4 nM final concentration) in AlphaScreen Buffer was mixed on the AlphaPlate by the dispenser. Then, the plate was incubated at 26 °C for 1 h. After incubation for 1 h, 10 µL of detection mixture was added, followed by further incubation at 26 °C for 1 h. The AlphaScreen signal was detected using an EnVision device with the AlphaScreen signal detection program (PerkinElmer).

For determination of IC50, the same assays were performed for GATA2 and GATA5 in the presence of various concentration of pyrrothiogatain (0 to 200 µM). The obtained data was fitted using the 4-parameter non-linear regression of GraphPad Prism version 8.0.

### Pyrrothiogatain (3-(2,5-dimethyl-1H-pyrrol-1-yl)thiophene-2-carboxylic acid)

Pyrrothiogatain (CAS No.: 477888-48-5) was purchased from Enamine (cat#EN300-51104) and Santa Cruz (cat#sc-352288A).

### Electrophoretic mobility shift assay

Electrophoretic mobility shift assays were performed as described previously^20^. In brief, four GATA family proteins (GATA2 to GATA5) were incubated with a ^32^P-labelled GATA-binding DNA probe for 20 min at 4 °C in the presence or absence of pyrrothiogatain (0 to 100 µM). The DNA-protein complex was separated from free oligonucleotides on 4% polyacrylamide gels, and then, the ^32^P-labelled DNA probes were detected using the Typhoon FLA 7000 (GE Healthcare).

### MTS assay

Jurkat cells were cultured at 37 °C with 5% CO_2_ in RPMI 1640 Medium (cat#72400047; Thermo Fisher) in the presence or absence of pyrrothiogatain (0 to 100 µM). Cell viability was measured using a CellTiter 96^®^ AQueous One Solution Cell Proliferation Assay (cat#G3582; Promega) according to the manufacturer’s protocol.

### Luciferase reporter assay

The IL-5 promoter activity was determined as previously described^22,23^. In brief, HEK293T cells were co-transfected with a firefly luciferase reporter (pGL3-IL-5 promoter), a renilla luciferase plasmid, and an expression vector (pcDNA3.1) using TransIT-LTI Regent (Cat#MIR2304, Mirus). Six hours after transfection, cells were treated with pyrrothiogatain (0 to 30 µM) and PMA (30 ng/mL) for 18 hours. The luciferase activity was measured using a Dual-Luciferase Reporter Assay System (cat#E1910, Promega).

### Immunoprecipitation

Co-immunoprecipitation analysis of GATA3 and SOX4 was conducted as follows. Immunoprecipitation of FLAG-tagged GATA3 in the presence of pyrrothiogatain (0 to 100 µM) was performed by mixing recombinant FLAG-GATA3 and AGIA-SOX4 with 10 µL of anti-FLAG M2 affinity agarose gel (cat#A2220, Sigma-Aldrich) by rotation at 4 °C for 2 h. The agarose gels were washed three times, and then the immunoprecipitated protein was detected by immunoblotting using anti-FLAG M2 antibody and anti-AGIA-HRP^36^.

### CD4^+^ T cell–differentiation cultures

C57BL/6 mice were purchased from Clea (Clea Japan, Inc., Tokyo, Japan). Total CD4^+^ T cells isolated from mouse spleen were prepared using a FITC anti-mouse CD4 antibody (Cat#100510, RM4–5, BioLegend) and anti-FITC-microbeads (Cat#130-097-050, Miltenyi Biotec). Naive CD4^+^ T cells (CD44^low^, CD62L^high^) were prepared using a MojoSort Mouse CD4 T cell Isolation Kit (Cat#480033), BioLegend). Biotinylated anti-CD44 (Cat#103004, IM7) and anti-CD25 antibodies (Cat#102004, PC61) were purchased from BioLegend. CD4^+^ T cells (7.5 × 10^5^) were stimulated using immobilized anti-TCR-β mAb (3 µg/mL, H57–597, BioLegend) plus an anti-CD28 mAb (1.0 µg/mL, cat#40–0281, TONBO Biosciences) for 2 days, and then, the cells were further expanded under similar conditions for an additional 3 days. IL-2 conditions: IL-2 (10 ng/mL, Pepro Tech); Th2 conditions: IL-2 (10 ng/mL, Pepro Tech), IL-4 (1 ng/mL, Pepro Tech), and anti-IFN-γ mAb (2.5 µg/mL, cat#40–731, TONBO Biosciences). Th1 conditions: IL-2 (10 ng/mL, Pepro Tech), IL-12 (1 ng/mL, Pepro Tech), and anti-IL-4 mAb (2.5 µg/mL, cat#40–731, TONBO Biosciences). The cultured cells were subjected to intracellular staining, ELISA assay, and RT-PCR. All animal experiments received approval from the Ehime University Administrative Panel for Animal Care. All animal care was conducted in accordance with the guidelines of Ehime University.

### Intracellular staining of cytokines and transcription factors

The *in vitro* differentiated CD4^+^ T cells were stimulated using an immobilized anti-TCR-β mAb (3 µg/ml, H57–597, BioLegend) for 6 h in the presence of monensin (2 µM). The cells were fixed with 4% paraformaldehyde (Cat#163–20145, Wako) and permeabilized with permeabilization buffer (50 mM NaCl, 5 mM EDTA, 0.02% NaN_3_, and 0.5% Triton X-100). Then, the cells were stained using the following antibodies, anti-IL-4-PE (Cat#504103, BioLegend, 1:50), anti-IL-5-APC (Cat#504305, BioLegend, 1:50), anti-IL-13-PE (Cat#12–7133–41, eBiosience, 1:50), anti-IFN-γ-FITC (Cat#562019, BD Biosciences, 1:500), and anti-IL-2-APC (Cat#503809, BioLegend, 1:50). For the intracellular staining of Gata3, the Foxp3/Transcription Factor Staining Buffer Kit (cat#TNB-0607, TONBO) was used according to the manufacturer’s protocol. Flow cytometry was performed using a Gallios Flow Cytometer instrument (Beckman Coulter) and a FACS Caliber instrument (BD Biosciences) and the results were analysed using the FlowJo software program (Tree Star, Ashland, OR, USA).

### ELISA assay

The CD4^+^ T cells cultured for five days were stimulated using an immobilized anti-TCR-β mAb (3 µg/mL, H57–597, BioLegend) for 16 h, and the culture supernatants were recovered. The amount of cytokines in the recovered supernatants was determined with ELISA using the following antibodies and reagents: anti-IL-4 mAb (Cat#554387, BD Biosciences), biotin-anti-IL-4 mAb (Cat#554390, BD Biosciences), anti-IL-5 mAb (Cat#554393, BD Biosciences), biotin-anti-IL-5 mAb (Cat#554397, BD Biosciences), anti-IFN-γ mAb (Cat#551216, BD Biosciences), biotin-anti-IFN-γ mAb (Cat#554410, BD Biosciences), anti-IL-2 mAb (Cat#554424, BD Biosciences), biotin-anti-IL-2 mAb (Cat#554426, BD Biosciences), streptavidin horseradish peroxidase (Cat#434323, Invitrogen), and TMB peroxidase EIA substrate kit (Cat#1721066, BioRad). For IL-13, the mouse IL-13 Duoset ELISA (Cat#DY413, R&D systems) was used for detecting the levels of the IL-13 cytokine. All antibodies and reagents were used according to the manufacturer’s protocols.

### Quantitative RT-PCR

Total RNA was isolated using the TRI Reagent (Molecular Research Center, Inc.). Reverse transcription and qRT-PCR were performed using the Transcriptor First Strand cDNA Synthesis Kit (Cat#04379012001, Roche) and FastStart Essential DNA Green Master (Cat#06402712001, Roche), according to the manufacturer’s protocols. qRT-PCR primers were used as follow; IL-4: 5′-GATCGGCATTTTCAACGAG-3′ (forward), 5′-CGAGCTCACTCTCTGTGGTG-3′ (reverse); IL-13: 5′-ACCCAGAGGATATTGCATGG-3′ (forward), 5′-TGGGCTACTTCGATTTTGGT-3′ (reverse); IFN-γ: 5′-ATCTGGAGGAACTGGCAAAA-3′ (forward), 5′-TTCAAGACTTCAAAGAGTCTGAGGTA-3′ (reverse); IL-2: 5′-GCTGTTGATGGACCTACAGGA-3′ (forward), 5′-TTCAATTCTGTGGCCTGCTT-3′ (reverse); Gata3: 5′-TTATCAAGCCCAAGCGAAG-3′ (forward), 5′-TGGTGGTGGTCTGACAGTT-3′ (reverse); and Tbx21: 5′-TCAACCAGCACCAGACAGAG-3′ (forward), 5′-AAACATCCTGTAATGGCTTGTG-3′ (reverse).

### Chromatin immunoprecipitation assay

The Magna ChIP Chromatin Immunoprecipitation Kit was used for the ChIP assay according to the manufacturer’s protocol (Merck-Millipore). Anti-GATA3 antibody (Cat#AF2605, R&D systems) was used for chromatin immunoprecipitation. The specific primers for the Th2 cytokine gene locus and the Roche Universal probes were used as follows: the V_A_ site in the IL-4 enhancer: 5′-GCCTGTTTCCTCTCAGCATT-3′ (forward), 5′-TGATAAAAGTGACTTGAAGGTTGG-3′ (reverse), probe #4; IL-4 IE: 5′-CCCAAAGGAGGTGCTTTTATC-3′ (forward), 5′-AAATCCGAAACTGA GGAGTGC-3′ (reverse), probe #75; CGRE: 5′-CTCTCCTGGTGGCGTGTT-3′ (forward), 5′-CTTTGCGCACCCTTGAAC-3′ (reverse), probe #53; and IL-5p: 5′-TCACTTTATCAGGAATTGAGTTTAACA-3′ (forward), 5′-GATCGGCTTTTCTTGAGCAC-3′ (reverse), probe #43;

### Statistical analysis and number of replicates per experiments

Statistical significance was calculated using two-tailed unpaired Student’s t-test (Microsoft Excel, USA). Statistical significance was accepted at p < 0.05. All *in vitro* assays were repeated at least three times.

### Data availability

All data generated or analysed in this study are included in this published article and its supplementary information files.

## Supplementary information

Supplementary Information
