# Supplementary material for: Pyrrothiogatain acts as an inhibitor of GATA family proteins and inhibits Th2 cell differentiation in vitro
Source: Sci Rep. 2019 Nov 22;9:17335. doi: 10.1038/s41598-019-53856-1 (PMC6874683; doi:10.1038/s41598-019-53856-1)
Supplement: Supplementary file 1 — Supplementary Information [file 41598_2019_53856_MOESM1_ESM.docx]

Pyrrothiogatain acts as an inhibitor of GATA family proteins and inhibits Th2 cell differentiation *in vitro*

Shunsuke Nomura^1^, Hirotaka Takahashi^1^, Junpei Suzuki^2^, Makoto Kuwahara^2^, Masakatsu Yamashita^2^ and Tatsuya Sawasaki^1*^

^1^Proteo-Science Center (PROS), Ehime University, 3 Bunkyo-cho, Matsuyama, Ehime 790-8577, Japan, ^2^Department of Immunology, Graduate School of Medicine, Ehime University, Shitsukawa, Toon, Ehime, Japan

*Corresponding Author:

Tatsuya Sawasaki

Proteo-Science Center, Ehime University, Matsuyama 790-8577, Japan

Tel: 81-89-927-8530

Fax: 81-89-927-9941

*E-mail:* [*sawasaki@ehime-u.ac.jp*](mailto:sawasaki@ehime-u.ac.jp)*.*

**Supplementary Method and Supplementary Figures 1 - 5**

**Supplementary Method**

**OVA-induced allergic airway inflammation model**

C57BL/6 mice at 7 weeks old were immunized intraperitoneally with 100 µg OVA (Cat#A5503, Sigma) in 2 % aluminium hydroxide gel (Cat#vax-alu-250, InvivoGen) on day 0. The mice were challenged intranasally with 100 µg OVA in saline on days 7, 8 and 9. Pyrrothiogatain was administrated intraperitoneally at a concentration of 20 mg/kg in PBS every day from day 0 to 6. The day after the last OVA challenge, BAL fluid cells were subjected to histological examination using the Diff Quick stain (Cat#16920, Sysmex). All animal experiments received approval from the Ehime University Administrative Panel for Animal Care. All animal care was conducted in accordance with the guidelines of Ehime University.

**Supplementary Figure S1: Pyrrothiogatain inhibited Th2 cell differentiation under IL-2 condition.**

(**A**) Intracellular staining of IL-4/IFN-γ (upper panel), IL-5/IL-13 (middle panel), and IL-2/IFN-γ (lower panel) in CD4+ T cells cultured under IL-2 conditions in the presence or absence of pyrrothiogatain (30 µM) for five days.

(**B**) Cytokine production induced in pyrrothiogatain-treated Th2 cells shown in panel (A) was determined by ELISA.

(**C**) Quantitative RT-PCR analysis of the pyrrothiogatain-treated Th2 cells shown in panel (A).

(**D**) Intracellular staining of GATA3 in CD4+ T cells stimulated with immobilized anti-TCRβ mAb plus anti-CD28 mAb under IL-2 conditions in the presence or absence of pyrrothiogatain (30 µM) for two days.

**Supplementary Figure S2: The effect of Pyrrothiogatain for Th1 cell differentiation.**

(**A**) Intracellular staining of IL-2/IFN-γ (lower panel) in naive CD4^+^ T cells cultured under Th1 conditions in the presence or absence of pyrrothiogatain (80 µM) for five days.

(**B**) Cytokine production induced in the pyrrothiogatain-treated Th1 cells shown in panel (A) was determined by ELISA.

**Supplementary Figure S3: OVA-induced airway inflammation model.**

(**A**) A schematic diagram of the experimental protocol of the OVA-induced airway inflammation model.

(**B**) The cell count of eosinophils (Eos.), neutrophils (Neu.), lymphocytes (Lym.), macrophages (Mac.), and total cells in BAL Fluid of the model mice shown (n = 5 per group). There was no significant difference between the vehicle control and pyrrothiogatain-administered mice, as detected by ANOVA and Tukey-test.

**Supplementary Figure S4: Uncropped blot images.**

**Supplementary Figure S5: Uncropped blot images.**
